# Understanding identity development in context: comparing reflective and situated approaches to identity

**DOI:** 10.3389/fpsyg.2024.1467280

**Published:** 2025-01-08

**Authors:** Mandy A. E. Van Der Gaag, J. Ole H. Gmelin, Naomi M. P. De Ruiter

**Affiliations:** ^1^Department of Developmental Psychology, Faculty of Behavioral and Social Sciences, University of Groningen, Groningen, Netherlands; ^2^Department of Theory and History of Psychology, Faculty of Behavioral and Social Sciences, University of Groningen, Groningen, Netherlands; ^3^University College Groningen, University of Groningen, Groningen, Netherlands

**Keywords:** identity, context, reflective identity, situated identity, complex dynamic system

## Abstract

Many recent approaches to identity share a foundational similarity with ecological psychology, namely, to place identity in its context. That is, they explicitly place identity in its physical and social environments. Yet, we can distinguish at least two different approaches that diverge fundamentally with regards to the role that this “context” has in identity. We refer to these approaches as “reflective identity” and “situated identity” approaches. While the reflective-identity approach views context and individual as separate entities with a bi-directional relationship, the situated-identity approach views context and individual as inherently intertwined and inseparable. While these approaches have emerged as independent from each other, we see potential for these two approaches to become comprehensively coordinated. To set the stage for such a coordination in future research, we provide a short overview of these different approaches to identity and describe where they align and diverge with regards to viewing identity as contextualized. After providing an overview of the key differences and similarities, we outline a possibility for integration and offer ideas for future lines of research that we see as fruitful for developing a comprehensive and coordinated approach to identity that takes context seriously.

## Introduction

Most identity researchers would agree that identity development takes place within particular socio-cultural contexts. Indeed, one of the most important founders of identity theory, Erikson (1956), emphasized that the individual’s sense of identity is always shaped by their social and historical context. Since Erikson, two main approaches to identity have emerged that take the task of placing identity in its socio-cultural context as central. We refer to these broad approaches as *reflective-identity* approaches and *situated-identity* approaches.

**Figure 1:**
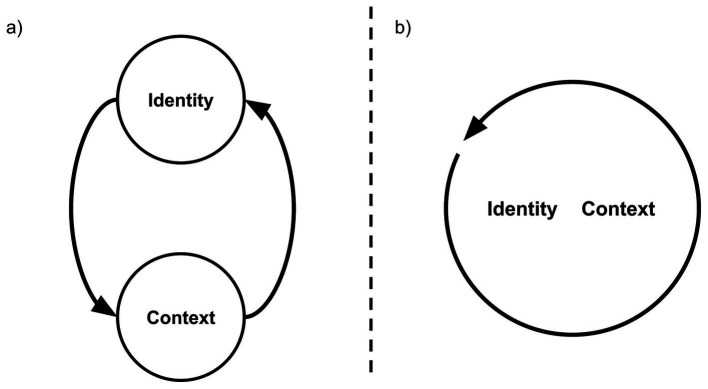
**(A)** The conceptualization of identity and context as distinct entities, with bi-directional influence (represented by the two arrows) within the reflective-identity approach. **(B)** The conceptualization of identity and context as continuously intertwined (represented by the circle-arrow) within the situated-identity approach.

Despite the fact that these approaches to identity share a dedication to understanding identity in context, we will show that the situational and reflective approaches have largely divergent methods and conceptual grounding (see also Antaki et al., 1996). Specifically, the reflective-identity approaches conceptualize identity as an inherently *individual* phenomenon, and view the context as critical yet inherently separate from an individual’s identity (De Ruiter and Gmelin, 2021; Lichtwarck-Aschoff et al., 2008; see Figure 1A). In contrast, situational-identity approaches conceptualize identity as a *social* phenomenon, meaning that it is constructed and enacted in the interaction between an individual and their context (Antaki and Widdicombe, 2008; Korobov, 2010). From this, an individuals’ identity and their context are viewed as fundamentally inseparable (Wetherell, 2007; see Figure 1B). Methodological differences have emerged from these conceptual differences. Where reflective-identity approaches study individuals as the unit of analysis, situational approaches study conversational practices as the unit of analysis (Gmelin and Kunnen, 2021; Wetherell, 2007).

In this mini-review, we provide an overview of these two approaches to identity, including their conceptual and methodological differences and similarities. With this, our aim is to clarify how these reflective and situated approaches to identity relate to one another, and develop priorities for future identity researchers interested in studying identity in context, so that we can establish a comprehensive and coordinated research agenda.

## Reflective-identity approach: context outside of person

The reflective-identity approach has been the dominant approach to identity within developmental psychology. It views identity as a self-reflective phenomenon, shaped in interaction with the context.

There are different approaches to the conceptualization and study of reflective identity such as narrative approaches to identity (see McAdams, 1993; McAdams and McLean, 2013) or the dialogical self theory (see Hermans, 2001). In the following, our review will focus on the identity-status approach (Marcia, 1966) which has been the most widely adopted approach within developmental psychology and which explicitly includes conceptualizations of identity development in context. There, identity is conceptualized as an individual’s collection of commitments that ideally develop through processes of active exploration (Kroger and Marcia, 2011).

*Commitments* are a key concept in the identity-status approach and have been operationalized in various ways, resulting in various conceptualizations of what it is exactly that one commits to (Van der Gaag et al., 2017; Waterman, 2015). This ranges from commitments to abstract self-views (typically assessed with interviews) to commitments to specific contexts or people (typically assessed with questionnaires). To illustrate, a commitment in the domain of career could be “I want to pursue a career as a lawyer.” Such commitments are assumed to be accessible to the individual through reflection, verbalizing the content of their commitments and indicating their strength. Thus, an understanding of identity in this framework relies heavily on how individuals experience and reflect on themselves, and how capable they are of reporting these reflections.

Identity processes have been studied in terms of varying amounts of exploration (both in-depth and in breadth), which enables the making and reconsidering of commitments, and in terms of the content of identity commitments (see Galliher et al., 2017; McLean et al., 2016; see Branje et al., 2021 for a review). Identity exploration, such as trying out a commitment or searching for information regarding possible commitments, is traditionally seen as a main driving force of commitment development (Marcia, 1966). If a strong commitment is formed through active exploration, the individual is considered to have an *achieved* identity status (Marcia, 1966), which is the most optimal of four identity statuses for one’s general psycho-emotional development (Kroger and Marcia, 2011).

While initial research in the identity status approach focussed on classifying individuals in different statuses, theorists such as Berzonsky (1992), Breakwell (1988), Grotevant (1987), Kaplan and O’Connor (1993), Kerpelman et al. (1997), and Kroger (1993) directed the field’s focus toward understanding the process of identity development and the role of context. Drawing from these theorists, Bosma and Kunnen (2001) advanced theory on identity development, by conceptualizing identity as a dynamic process of continuous interaction between individual and context.

In Bosma and Kunnen’s (2001) dynamic model of identity, the individual interacts with the context and encounters information that is either in line with or in conflict with their identity. When conflict arises, potential for identity development follows. This model of identity thus prioritizes the role of everyday contexts, and highlights the resulting process of accommodation (i.e., adjusting identity to new contextual information to deal with internal conflict) and assimilation (i.e., ignoring or reinterpreting contextual information to deal with internal conflict) as critical cognitive mechanisms utilized to make sense of the experiences that these contexts evoke. Building on this view, reflective-identity studies have investigated the ways in which identity development is contingent on everyday contexts, considering day-to-day or week-to-week changes in identity processes (for a review, see Branje et al., 2021). Methodologically, this approach entails the use of short questionnaires that allow for the repeated assessment of a small set of variables (e.g., Becht et al., 2017; Klimstra et al., 2010; Van der Gaag et al., 2016).

We wish to highlight the conceptual underpinnings of this approach. First, from both the focus on commitments as the key operationalization of identity, and the methodological focus on individual’s self-reflections and self-report, it is evident that identity is approached as an individual and reflective phenomenon. Second, this approach stresses that identity exists independent of contexts, such that identity exists before and outside an encounter with a context, and the context exists outside of the individual (Antaki et al., 1996). The idea is that the context evokes an experience that the individual must make sense of (i.e., assimilation or accommodation). Thus in this approach the context is seen as a variable that is separate from identity, but does have a critical relationship with identity.

Several studies within this approach have sought to understand how this relationship between identity and context unfolds. For instance via the experiences or moods that the context evokes. Klimstra et al. (2016) showed that daily mood relates to commitment strength and exploration dynamics, and Van der Gaag et al. (2017), showed that weekly commitment strength dynamics are affected by emotional experiences (both positive and negative) elicited by daily contexts. Interpersonal relationships are often examined as a critical context in which identity develops. For instance Beyers and Goossens (2008) show a bi-directional relationship: parental behaviors (such as warm and supportive parenting) predict whether adolescents develop a healthy sense of self, and evaluative phases of identity formation (i.e., exploration in depth and commitment identification) predict more supportive parenting. Similarly, Crocetti et al. (2017) show that, while supportive parenting predicts healthy development of self in adolescents, this in return helps to improve family relationships. More broadly, the role of context as a socio-cultural dimension is also considered important for identity development. For example, McAdams (2001) shows that culture transmits notions of which experiences are in fact meaningful for identity. Thus these studies aim to show how context and identity are bi-directionally influenced as separate variables.

What these examples illustrate is that this approach considers the context as having a crucial bi-directional relationship with identity development, but that it is also separated from the individual’s identity, both conceptually and methodologically. The context is conceptualized in terms of information that elicits (emotional) responses in the individual (i.e., an identity experience), and can result in changes in identity commitments. This conceptualization is made explicit in the landscape of identity model (Van der Gaag et al., 2020) which defines identity as a system consisting of an individual’s commitments and processes of exploration, while the context is understood as a separate system that is selected by the individual and that elicits identity-related experiences in the identity system. Besides this conceptual separation, the separation between an individual’s identity and their context is also often reflected in the methodology employed. For instance, aspects of contexts (e.g., an emotional experience elicited by context) and identity (e.g., commitment strength) are operationalized as variables with (causal) associations that can be mapped statistically (for a more extensive analysis, see also De Ruiter, 2023).

## Situated-identity approach: person-context as inherently inseparable

While reflective-identity approaches have traditionally dominated (developmental) psychology, psychologists are increasingly adopting a more situated approach to identity. Drawing on a diverse set of theoretical perspectives such as discursive psychology (Edwards and Potter, 1992), socio-linguistics (Antaki, 2012; Stokoe, 2012), and relational approaches (Carpendale and Lewis, 2018) these researchers have (often explicitly) pushed against individualized and reflective approaches to identity. Instead, they view identity as “something that people do which is embedded in some other social activity, and not something they ‘are’” (Antaki and Widdicombe, 2008, p. 191).

In the situated approach, the individual’s identity and context are understood as inherently intertwined: here identity is a social rather than an individual phenomenon. As such, situated-identity approaches challenge the core assumption held in reflective approaches regarding context as separate from identity, and thus also the empirical goal of determining cause-effect relationships between context and identity (Mascolo and Bidell, 2020). Specifically, the situated approach critiques the treatment of contexts as discrete *external* variables that carry information for the *internal* identity system to interpret, and which may elicit or evoke a response from the individual (e.g., Overton and Lerner, 2014), or which may lead to a reinforcement, weakening or reorganization of the internal identity structure. From a situated-identity perspective this understanding of identity and context is problematic for several reasons (for an in-depth discussion see Van Geert and De Ruiter, 2022). For example, it does not provide complete information about the situated behaviors and actions that promote identity formation (e.g., Gmelin and Kunnen, 2021), while such information is required for practitioners wishing to stimulate an individual’s identity development.

Instead, in the situated approach, context is understood as an inherent part of the identity system, offering the individual various “opportunities for action” (i.e., *affordances*; Overton and Lerner, 2014). Such opportunities for action typically include other people, locations and spaces, objects and artifacts, as well as tasks and activities. From this starting point, situated approaches argue that the person and their changing context should be viewed as co-constructing each other as *one* person-environment system (Carpendale and Lewis, 2018; Hollway, 2008). For example, social interactions can be seen as sequences of offers between conversation partners, ultimately constructing agreements about who the conversational partners are to each other in the larger cultural context. This approach does not understand context as carrying information, but as the site for joint meaning-making, and an arena for navigating and negotiating which bits of information are meaningful (Stokoe, 2010). The empirical goal from this approach is then to describe active dynamics between the person and the environment and the resulting unfolding of meaning and identity construction.

Within the situated approach to identity, the process of meaning making is an intersubjective endeavor (Cipolletta et al., 2022). This means that rather than viewing identity construction as an internal process of interpreting and integrating information from *external* contexts, identity construction is viewed as a shared process between the person and their engagement *with* their contexts (i.e., meaning is co-constructed; De Jaegher and Di Paolo, 2007; Fogel, 1993). For example, Anderson (2009) showed how students use material artifacts such as self-evaluation questionnaires for a group activity as a resource for jointly positioning one of their peers as the kind of person who is a “slow” learner. Because this process of co-constructing identities often takes place in social interactions (whether real or imagined), conversations are the most common context studied within the situated-identity approach.

An example of a situated approach that relies on the idea of joint construction of meaning making is the *discursive positioning* approach, which commonly draws on analyses of actions situated in everyday contexts and conversations (Bamberg, 1997; Gmelin and Kunnen, 2021; Korobov, 2010). This approach draws from positioning theory, and examines how individuals and groups assign roles and identities to themselves and others in the context of interactions. This approach stresses the constructive nature of identity, where positions are seen as the “effect that certain discursive actions have for establishing the identities of the participants” (Korobov, 2010; p. 269), where discursive actions include speech acts (e.g., requesting information, giving instructions, or complaining; Korobov, 2010).

From this standpoint, identity experiences and behavior are not considered to be reflections of an individual’s internal identity. Rather, identity positions are assumed to be social tools that emerge while individuals engage in the local social-business of the interaction (Breakwell, 1986; Korobov, 2010). For example, they may serve to establish authority or resolve a conflict. To illustrate, Gmelin and Kunnen (2021) demonstrated how two participants in a conversation on sex, in the course of negotiating who should be the first to disclose intimate information in their specific interaction, positioned themselves as “not giving a fuck” (i.e., an identity position) about sharing intimate information in general. These positions served as tools in the negotiation process, allowing participants to justify their reluctance to disclose first while maintaining an image of aloofness.

The previous examples thus show that from a situated-identity approach, the identity system is defined as the person and context. This has been made explicit by the dynamic systems model of role identity (Kaplan and Garner, 2017). Similar to the landscape of identity model (van der Gaag et al., 2020), this model of identity also relies on dynamic systems principles, but it describes a different system: they do not focus on the commitments within the individual, but rather on the individual occupying a role (e.g., student, friend) contingent on a given social-cultural context (e.g., a particular school). The individual and social-cultural context continuously interact, and this interaction determines the action and experience possibilities of the individual: one specific social-cultural context may afford very different actions and experiences than another context.

Thus, contrasting the reflective-identity approach, we see that the situated-identity approach considers individuals as actively co-constructing identity with contexts, creating one intertwined process. As a consequence, the situated approach conceptualizes experiences as an individual’s active engagement with the immediate environment in the context of socio-cultural constraints, rather than in terms of emotional and evaluative responses *elicited* by an external context.

## Discussion: toward a comprehensive and coordinated approach to identity-in-context

As we have shown above, the reflective-identity and situated-identity approaches have a fundamentally similar objective: to understand identity as a dynamic and ongoing process that is situated in contexts, moving the field beyond a de-contextualized approach to identity. Despite their shared aim and contribution, these two broad approaches to identity differ in their core conceptualizations of the relationship between individual and context. The difference can be described in terms of how the identity “system” is defined. In reflective-identity approaches, the identity system is defined as a pattern of self-views within the individual, which has a bi-directional relationship with the external context. In situated-identity approaches, the identity system is defined as a pattern of meanings that emerges between an individual and their context.

A complex dynamic systems approach may provide a framework for how to integrate these different conceptions of the identity “system.” From a dynamic systems approach, researchers must necessarily define the boundaries of a system before it can be studied, which will depend on the phenomenon that we wish to study (De Ruiter et al., 2019). As De Ruiter and Gmelin (2021) stressed, the outlined approaches may represent attempts to understand different *features* of identity: situated actions and experiences (situated-identity approach) versus cognitive self-representations (reflective-identity approach). Both of these features can exist side by side and develop across both the short term and the long term. De Ruiter and Gmelin’s work encourages us to view these features in a pluralistic and non-competitive way and sets the stage for a critical question in identity research, namely, how are these two features of identity related to one another? This is a major gap in identity research, which we believe should be a priority in the identity-research agenda.

**Figure 2:**
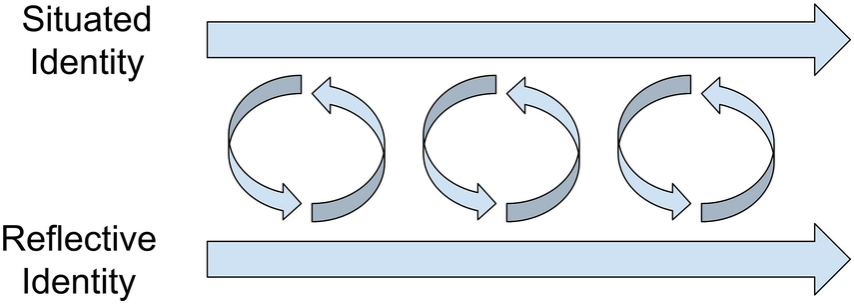
Hypothesized interaction between two identity systems over time: situated identity (top) and reflective identity (bottom).

Approaching these two features of identity as interconnected systems that develop in parallel to each other, allows us to consider the developmental dynamics between them. Reflective and situated features of identity can be understood as parallel (i.e., non-hierarchical) components of a larger system that feed into each other in a cyclical manner (i.e., they exhibit horizontal causality; Van Geert and De Ruiter, 2022; see also Figure 2). This interconnected developmental process may involve the reflection upon our emerging action patterns, in such a way that an increasingly consistent reflective identity may emerge over the long term. Simultaneously, these long-term reflective features of identities may start to play a role in how we present ourselves in certain contexts, thus constraining our situated identity actions.

Interestingly, this line of reasoning allows for the possibility that reflective-identity features are well aligned with situated-identity features, but they may also be qualitatively distinct. They may be distinct, for example, for individuals who are not concerned with self-reflection or who have poor reflective or meta-cognitive capabilities. In such cases, we can imagine that patterns of situated-identity features may emerge over time without any self narrative. Alternatively, emergent self narratives may be inconsistent with situated identity behavior and may be minimally directive for how the individual will experience themselves in concrete contexts. Such hypothetical discrepancies between situated and reflective identity, and their possible consequences, form an interesting area for future research.

Indeed, future research should focus on how the dynamic interplay between reflective and situated features of identity occurs within contexts, and how individuals differ in this process. For example, the same event may be a powerful identity changing experience for one individual, but an insignificant and mundane experience for the next individual. Furthermore, because both situated and reflective approaches share an interest in studying processes over time, their differing methodologies offer opportunities for understanding the dynamic interplay between reflective and situated features. The reflective identity approach tends to analyze longitudinal questionnaire or diary data, while the situated-identity approach often studies verbal and behavioral interaction partners, for example during a conversation. Future research should prioritize linking these methods, for instance, by integrating them within a single longitudinal study to compare the unique insights each provides.

Finally, a brief note is in order on the limitations of this paper. Even though we have decided to characterize literature on identity in two types of approaches, this is not an exhaustive review. Certainly views on identity have been omitted that could be relevant to include, but for the sake of clarity and simplicity, we have decided to limit ourselves to include only exemplary, but important, samples of the literature.

## Conclusion

The role of context in identity has been taken seriously by both the reflective-identity approach and the situated-identity approach, but both have fundamentally different conceptions of the role of the context. While the reflective identity approach views the context as bi-directionally related to, but separate from, the individual, the situated identity approach views context and individual as inherently intertwined and inseparable. We have proposed that these approaches may be understood as the study of two different identity systems that interact over time: situated identity-experiences shape reflective identity, and this reflective identity then constrains situated identity-experiences. This leads to interesting questions related to how these systems interact, including how they come to be coherent or discrepant. Pursuing future research from a combination of these two approaches to identity in context might be one of the most interesting paths forward to understand how identity develops.
